# Inflammatory Bowel Diseases Are Not Associated with an Increased Risk of Autoimmune Thyroiditis

**DOI:** 10.3390/medsci14010065

**Published:** 2026-01-31

**Authors:** Alexander Barton, Christian Labenz, Stephan Grabbe, Jörn M. Schattenberg, Leonard Kaps, Karel Kostev

**Affiliations:** 1 Department of Medicine II, Saarland University Medical Center, Saarland University, 66421 Homburg, Germany; joern.schattenberg@uks.eu (J.M.S.); leonard.kaps@uks.eu (L.K.); 2 First Department of Medicine, University Medical Center of the Johannes Gutenberg-University, 55131 Mainz, Germany; christian.labenz@unimedizin-mainz.de; 3 Cirrhosis Center Mainz (CCM), University Medical Center of the Johannes Gutenberg-University, 55131 Mainz, Germany; 4 Department of Dermatology, University Medical Center of the Johannes Gutenberg-University, 55131 Mainz, Germany; stephan.grabbe@unimedizin-mainz.de; 5 Epidemiology, IQVIA, 60549 Frankfurt am Main, Germany; karel.kostev@iqvia.com

**Keywords:** inflammatory bowel disease, autoimmune thyroiditis, Graves’ disease, cohort study

## Abstract

**Background/Objectives**: The incidence of inflammatory bowel disease (IBD) is rising worldwide, particularly in Asia, while the highest prevalence remains in North America and Europe. Evidence on the relationship between IBD and the development of autoimmune thyroiditis is limited. This study investigated the association between IBD and a subsequent autoimmune thyroiditis in a large German primary care cohort over a 10-year period. **Methods**: Patients with IBD were propensity score matched to non-IBD individuals in a 1:5 ratio based on age, sex, index year, and average annual number of physician visits during follow-up. A total of 20,084 IBD patients—including 8791 with Crohn’s disease and 11,293 with ulcerative colitis—and 100,420 matched controls were included. The primary outcome was the cumulative incidence of autoimmune thyroiditis, including Hashimoto’s thyroiditis and Graves’ disease. The association between IBD and autoimmune thyroiditis was evaluated using univariable conditional Cox regression analysis. **Results**: In the overall cohort, no significant association was found between IBD (Crohn’s disease or ulcerative colitis) and autoimmune thyroiditis (Hashimoto’s or Graves’ disease). However, among patients aged ≥ 65 years, IBD was associated with a significantly increased risk of Graves’ disease (HR 2.83; 95% CI 1.56–5.15), an effect observed in both Crohn’s disease (HR 3.23; 95% CI 1.20–8.69) and ulcerative colitis (HR 2.64; 95% CI 1.25–5.60). **Conclusions**: While IBD was not associated with autoimmune thyroiditis overall, a significant positive association with Graves’ disease was observed among patients aged ≥ 65 years, highlighting the importance of age-specific risk assessment.

## 1. Introduction

Inflammatory Bowel Disease (IBD), comprising Crohn’s disease (CD) and ulcerative colitis (UC), is a chronic, immune-mediated disorder of the gastrointestinal tract characterized by relapsing inflammation and clinical manifestations such as diarrhea, abdominal pain, and weight loss [1]. Globally, more than 6.8 million individuals are affected by IBD, with incidence rates continuing to rise, particularly in newly industrialized regions such as Asia [2]. Extraintestinal manifestations (EIM) in patients with IBD are frequent, while CD patients generally have a higher prevalence of EIM (up to 35–55%) compared to UC (19–35%), including musculoskeletal, dermatological, hepatobiliary, and endocrine involvement [3,4]. This reflects that IBD is a systemic autoinflammatory disease, affecting different organ systems and not exclusively restricted to the intestinal tract. In consequence, other autoimmune diseases occur more frequently in patients with IBD. Among these, liver-related autoimmune conditions are among the most strongly associated. The pooled prevalence of primary sclerosing cholangitis (PSC), a progressive disease characterized by chronic, fibrosing inflammation of the biliary system and associated with poor prognosis, among patients with IBD is approximately 2% (range 0.4–8%), with higher rates observed in ulcerative colitis (UC, ~2.5–4%) compared to Crohn’s disease (CD, ~0.6–1%) [5]. Conversely, the association is even more pronounced in the opposite direction: the majority of patients with PSC (60–80%) have concomitant IBD, predominantly UC (75–90% of PSC-IBD cases) [6]. In addition, other autoimmune conditions are observed more frequently in patients with IBD. These include joint and skin disorders such as rheumatoid arthritis, spondyloarthritis, and psoriasis, as well as neurological autoimmune diseases like multiple sclerosis and myasthenia gravis, which are less common but still occur at higher rates compared to the general population [7].

The association between IBD and endocrine disorders, such as Graves’ disease and Hashimoto’s thyroiditis, has been less extensively studied, particularly in large population-based cohorts, and available findings remain heterogeneous [8,9,10,11]. Hypothyroidism, in particular, appears to be associated with a more severe disease course in patients with IBD [12]. Early diagnosis of concomitant hypothyroidism, which typically manifests in middle-aged patients, and its timely treatment may therefore positively influence clinical outcomes.

Therefore, the aim of this study was to investigate whether IBD is associated with an increased risk of autoimmune thyroid disease using a large primary care cohort in Germany.

## 2. Materials and Methods

### 2.1. Database

This retrospective cohort study was based on the Disease Analyzer database (IQVIA), which contains anonymized data on prescriptions, diagnoses, and basic demographic and clinical characteristics derived directly from the electronic health records of general practitioners and specialists in Germany [13]. The database covers approximately 3000 outpatient practices nationwide and has been validated as representative of both general and specialized medical care in Germany [13]. It has also been widely used in previous studies investigating inflammatory bowel disease [14].

### 2.2. Study Population

**Figure 1:**
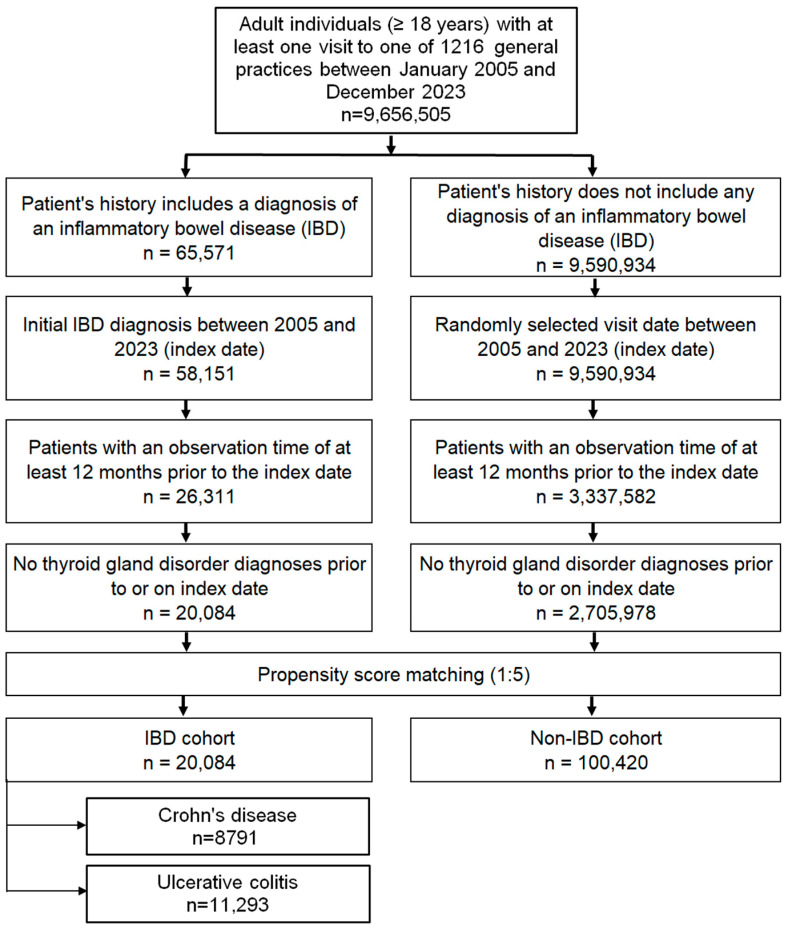
Selection of study patients.

The study included adult patients (≥18 years) with a first recorded diagnosis of CD (ICD-10: K50) or UC (ICD-10: K51) documented in 1 of 1216 general practices across Germany between January 2005 and December 2023 (index date; Figure 1). To minimize bias, only patients with at least 12 months of medical history prior to the index date were eligible, and those with documented thyroid disorders during this pre-index period were excluded.

A control cohort without IBD was created using nearest-neighbor propensity score matching in a 5:1 ratio. Matching variables included age, sex, index year, and average annual number of physician visits during follow-up. For controls, the index date was defined as a randomly selected consultation within the study period (2005–2023; Figure 1). Covariate balance between the IBD and non-IBD groups was assessed using the standardized mean difference (SMD), with values < 0.1 indicating adequate balance.

### 2.3. Study Outcomes

The primary outcomes were new diagnoses of Hashimoto’s thyroiditis (ICD-10: E06.3) and Graves’ disease (ICD-10: E05.0) within a follow-up period of up to 10 years after the index date, analyzed in relation to IBD status.

### 2.4. Statistical Analyses

Incidence rates for Hashimoto’s thyroiditis and Graves’ disease were expressed as cases per 100,000 person-years. Given the large number of sub-analyses and the relatively low cumulative incidence (<1% in most groups), Kaplan–Meier curves were not applied. Associations between IBD and thyroid diseases were estimated using univariable Cox proportional hazards models, stratified by IBD subtype (CD and UC), age group, and sex. Results are presented as hazard ratios (HRs) with 95% confidence intervals (CIs). To account for multiple testing across 18 models, a conservative significance threshold of *p* < 0.001 was applied. All analyses were conducted using SAS version 9.4 (SAS Institute, Cary, NC, USA).

## 3. Results

### 3.1. Basic Characteristics of the Study Sample

A total of 20,084 patients with IBD (CD: 10,264; UC: 9820) and 100,420 matched controls were included. Baseline characteristics after 1:5 propensity score matching are shown in Table 1 (for additional information see Table S1). The mean age of the overall cohort was 49.4 years (SD 18.2), and 48.8% were female. The distributions of age, sex, index year, and annual physician visits were nearly identical between groups, with standardized mean differences close to zero, indicating excellent covariate balance.

### 3.2. Incidence of Hashimoto’s Thyroiditis and Graves’ Disease

**Figure 2:**
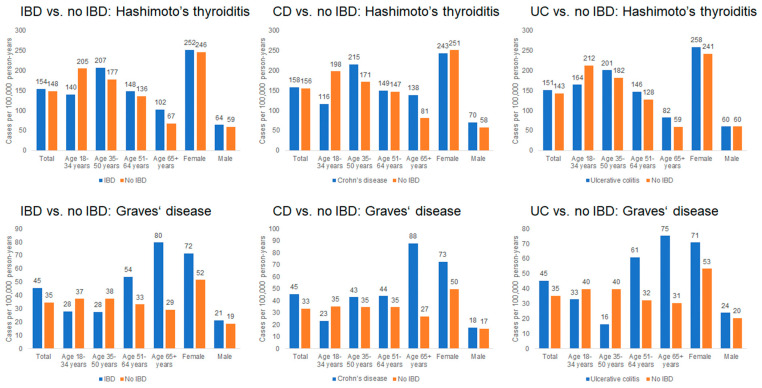
Incidence rates of Hashimoto’s thyroiditis and Graves’ disease in patients with and without IBD (per 100,000 person-years).

As shown in Figure 2, the incidence of Hashimoto’s thyroiditis was comparable between groups, with 154 cases per 100,000 person-years among IBD patients and 148 cases per 100,000 person-years among non-IBD patients. Incidence increased with age and was higher in women; for example, among women aged ≥ 65 years, the incidence was 252 cases per 100,000 person-years in the IBD cohort and 246 cases per 100,000 person-years in the non-IBD cohort.

The incidence of Graves’ disease was 45 cases per 100,000 person-years among IBD patients and 35 cases per 100,000 person-years among non-IBD patients. The highest incidence occurred in individuals aged ≥ 65 years, particularly among women (72 cases per 100,000 in IBD vs. 52 cases per 100,000 in non-IBD). In contrast, incidence among younger adults (<35 years) remained below 40 cases per 100,000 in both cohorts.

### 3.3. Association Between IBD and Hashimoto’s Thyroiditis

Cox regression results are shown in Table 2. Overall, IBD was not significantly associated with Hashimoto’s thyroiditis (HR 1.06; 95% CI 0.89–1.26), with similar findings for Crohn’s disease (HR 1.04; 95% CI 0.80–1.34) and ulcerative colitis (HR 1.08; 95% CI 0.86–1.36). No sex-specific associations were identified. A non-significant trend toward increased risk was observed in patients aged ≥ 65 years (HR 1.54; 95% CI 0.95–2.48), particularly among those with CD (HR 1.73; 95% CI 0.86–3.49).

### 3.4. Association Between IBD and Graves’ Disease

No overall association was observed between IBD and Graves’ disease (HR 1.32; 95% CI 0.96–1.83). In patients aged ≥ 65 years, however, IBD was associated with more than a twofold increased risk (HR 2.83; 95% CI 1.56–5.15). This age-related effect was present in both CD (HR 3.23; 95% CI 1.20–8.69) and UC (HR 2.64; 95% CI 1.25–5.60). No significant associations were detected in younger age groups or by sex.

## 4. Discussion

In this large cohort study of matched patients with and without IBD, we found no association between IBD and an increased risk of autoimmune thyroiditis, including Hashimoto’s thyroiditis and Graves’ disease, over a 10-year follow-up. However, there was a significant association between IBD, both UC and CD and Graves’ disease in patients older than 65 years. While earlier studies suggested that Graves’ disease might be more frequent in patients with IBD, more recent nationwide population-based cohorts, including a Danish registry study, did not confirm a clear association. In detail, an older single-center study from the UK reported an increased prevalence of Graves’ disease among UC patients compared with the general population, suggesting a possible link between UC and autoimmune thyroid disease [15]. In contrast, a Danish nationwide registry study did not observe an increased risk of Graves’ disease among more than 30,000 IBD patients, with risk estimates overlapping those of the general population [16]. There was also no increased risk for older patients. Consistent with our findings, the Danish study found also no increased risk of either Hashimoto’s thyroiditis in patients with IBD. A bidirectional Mendelian randomization study published in 2022 investigated the causal relationship between IBD and Graves’ disease [17]. The analysis demonstrated that a genetic predisposition to IBD was associated with a 24% increased risk of Graves’ disease (odds ratio [OR] 1.24, 95% confidence interval [CI] 1.01–1.52). Stratified analyses revealed disease-specific effects: CD was significantly associated with an elevated risk of Graves’ disease, whereas UC appeared to confer a protective effect. In the reverse direction, genetic liability to Graves’ disease was linked to a modest increase in the risk of CD, but not UC or overall IBD. The authors concluded that these findings may indicate a higher comorbidity burden between Graves’ disease and CD, but not between Graves’ disease and UC.

However, the biological mechanisms underlying the associations between IBD and Graves’ disease remain to be elucidated and warrant further investigation. Notably, vitamin D deficiency has been implicated in the onset and progression of several autoimmune diseases, including multiple sclerosis, IBD, and type 1 diabetes mellitus [18]. In an observational study, the vitamin D status in patients with and without remission of Graves’ disease has been assessed [19]. Serum vitamin D levels were significantly lower in Graves’ disease patients without remission than in those with remission. There is a well-established association between IBD and vitamin D deficiency. Multiple studies and meta-analyses consistently demonstrate that patients with IBD—including both CD and UC—are significantly more likely to be vitamin D deficient than healthy controls [20,21,22]. Compelling evidence indicates that lower vitamin D levels in patients with IBD are associated with higher disease activity, elevated inflammatory markers (e.g., CRP), more frequent flares, increased rates of hospitalization and surgery, and reduced quality of life. The evidence regarding the direct therapeutic effect of vitamin D supplementation on IBD activity remains inconclusive, with some studies demonstrating benefit while others report no significant effect [23,24]. Although the exact pathomechanisms of vitamin D deficiency in autoimmune diseases are not fully understood, the activation and regulation of T-cells appear to play a crucial role. Baeke et al. have shown that vitamin D exerts potent immunomodulatory effects and contributes to the pathogenesis of autoimmune diseases. Specifically, vitamin D inhibits the production of the Th1-polarizing cytokine IL-12, thereby indirectly shifting T-cell polarization from proinflammatory Th1 toward an anti-inflammatory Th2 phenotype. In CD4^+^ T-cell responses, vitamin D directly suppresses Th1 cytokines (IL-2 and IFN-γ) while enhancing the production of the Th2 cytokine IL-4 [18].

Given the observed association between inflammatory bowel disease (IBD) and Graves’ disease in older patients, it remains unclear whether advanced age itself represents a risk factor for disease onset. In IBD, approximately 10–20% of cases are diagnosed after the age of 60, and elderly onset IBD is increasingly recognized as a distinct and growing entity [25,26]. Nevertheless, the majority of IBD diagnoses occur in younger and middle-aged individuals between 20 and 40 years [27]. For Graves’ disease, however, there is no strong evidence to suggest that the incidence is higher in older adults. Most cases are diagnosed in younger to middle-aged patients, and the current literature does not support an increased risk of late-onset disease [17]. While the evidence does not indicate that IBD or Graves’ disease is more likely to present in the elderly, other autoimmune disorders such as rheumatoid arthritis and giant cell arteritis are well known to have higher incidence rates in this age group [28,29]. Moreover, in conditions like multiple sclerosis and systemic lupus erythematosus, later-onset disease is associated with more progressive clinical courses and greater disability [30,31,32]. Mechanistically, immune aging (immunosenescence) contributes to this increased risk by driving T- and B-cell dysfunction, promoting a pro-inflammatory state (“inflammaging”), and fostering the accumulation of age-associated immune cell subsets, which may facilitate the development of autoimmunity in older adults [32,33].

The study draws on the Disease Analyzer database, which offers a robust and representative sample of outpatient practices across Germany, enabling longitudinal analyses of real-world clinical data. Its strength lies in the breadth of routinely collected information, including different diagnoses and the capacity for long-term follow-up. The use of propensity score matching and stratified Cox regression enhances the methodological rigor by controlling for key confounders and allowing age- and sex-specific risk estimation. However, the database has notable limitations. It lacks clinical granularity, such as laboratory results and disease severity measures, which restrict insight into underlying mechanisms. Furthermore, it does not include hospital data, sociodemographic variables, or lifestyle factors, as well as vitamin D laboratory values, and relies solely on ICD-10 coding, which may introduce misclassification bias. Methodologically, the study is constrained by its reliance on the absence of time-to-event modeling, which was precluded by low incidence rates. Additionally, autoimmune thyroiditis diagnoses were not validated through laboratory confirmation, and prior thyroid disease was excluded, potentially underestimating associations. Importantly, because the index date was defined as the first IBD diagnosis, only baseline covariates could be included in the analyses; IBD-related therapies typically occur after cohort entry and could not be incorporated without introducing time-dependent bias. As a result, potential treatment-related effects could not be evaluated within this design. These limitations underscore the need for cautious interpretation of findings and highlight areas for future research using more comprehensive datasets.

Our findings indicate no positive associations between IBD and Hashimoto’s thyroiditis and Graves’ disease in the entire patient cohort. In contrast, older patients with IBD may be more susceptible to Graves’ disease, suggesting that regular thyroid function testing (via TSH measurement) in middle-aged patients could help positively influence the course of IBD. Furthermore, assessing vitamin D levels and providing supplementation when deficiency is documented is justified in patients with autoimmune disorders such as IBD, Hashimoto’s thyroiditis, and Graves’ disease.

## Figures and Tables

**Table 1:** Baseline characteristics of the study sample (after 1:5 propensity score matching).

Variable	Proportion Among Patients with IBD (N, %) N = 20,084	Proportion Among Patients Without IBD (N, %) N = 100,420	SMD
Age (Mean, SD)	49.4 (18.2)	49.3 (18.5)	0.002
Age 18–34 years	5280 (26.3)	26,400 (26.3)	
Age 35–50 years	5282 (26.3)	26,410 (26.3)	
Age 51–64 years	4901 (24.4)	24,505 (24.4)	
Age 65+ years	4.621 (23.0)	23,105 (23.0)	
Female	9806 (48.8)	49,030 (48.8)	0.000
Male	10,278 (51.2)	51,390 (51.2)	
Year of study inclusion			0.000
2005–2008	1858 (9.3)	9290 (9.3)	
2009–2012	3079 (15.3)	15,395 (15.3)	
2013–2016	4611 (23.0)	23,055 (23.0)	
2017–2020	5786 (28.8)	28,930 (28.8)	
2021–2023	4750 (23.6)	23,750 (23.6)	
Average number of physician visits per year	9.0 (4.5)	9.0 (4.5)	0.000

Proportions of patients in N, % given, unless otherwise indicated. SD: standard deviation. SMD: standardized mean difference.

**Table 2:** Association between IBD and subsequent diagnoses of Hashimoto’s thyroiditis and Graves’ disease in patients treated in general practices in Germany (univariable Cox regression models).

	IBD		Crohn’s Disease		Ulcerative Colitis	
Patient Subgroup	HR (95% CI)	*p* Value	HR (95% CI)	*p* Value	HR (95% CI)	*p* Value
**Hashimoto’s thyroiditis**						
Total	1.06 (0.89–1.26)	0.501	1.04 (0.80–1.34)	0.770	1.08 (0.86–1.36)	0.518
Age 18–34 years	0.68 (0.48–0.98)	0.040	0.59 (0.34–1.03)	0.061	0.77 (0.48–1.25)	0.297
Age 35–50 years	1.20 (0.91–1.58)	0.192	1.28 (0.85–1.93)	0.242	1.14 (0.79–1.65)	0.473
Age 51–64 years	1.12 (0.80–1.59)	0.508	1.08 (0.63–1.83)	0.789	1.17 (0.74–1.83)	0.507
Age 65+ years	1.54 (0.95–2.48)	0.078	1.73 (0.86–3.49)	0.127	1.40 (0.73–2.71)	0.312
Female	1.05 (0.86–1.27)	0.659	1.00 (0.75–1.33)	0.975	1.09 (0.84–1.41)	0.536
Male	1.12 (0.77–1.61)	0.563	1.23 (0.70–2.15)	0.473	1.04 (0.64–1.70)	0.876
**Grave’s disease**						
Total	1.32 (0.96–1.83)	0.092	1.35 (0.82–2.21)	0.241	1.30 (0.85–2.00)	0.225
Age 18–34 years	0.74 (0.33–1.66)	0.461	0.65 (0.19–2.22)	0.489	0.81 (0.27–2.40)	0.703
Age 35–50 years	0.72 (0.35–1.46)	0.357	1.21 (0.48–3.05)	0.679	0.39 (0.12–1.29)	0.122
Age 51–64 years	1.66 (0.91–3.02)	0.098	1.27 (0.46–3.46)	0.646	1.95 (0.92–4.13)	0.080
**Age 65+ years**	**2.83 (1.56–5.15)**	**<0.001**	**3.23 (1.20–8.69)**	**0.020**	**2.64 (1.25–5.60)**	**0.011**
Female	1.41 (0.97–2.05)	0.076	1.46 (0.84–2.55)	0.182	1.36 (0.82–2.26)	0.237
Male	1.11 (0.58–2.11)	0.756	1.00 (0.33–3.02)	0.998	1.17 (0.53–2.58)	0.697

## Data Availability

The data presented in this study are available on request from K.K. with the permission of IQVIA. The data are not publicly available due to legal restrictions.

## Supplementary Materials

The following supporting information can be downloaded at: https://www.mdpi.com/article/10.3390/medsci14010065/s1. Table S1. Number of events (M. Hashimoto, Grave’s disease) in individuals with and without IBD (Crohn’s disease, Ulcerative colitis).

## Abbreviations

The following abbreviations are used in this manuscript:

CD	Crohn’s disease
CI	Confidence interval
EIM	Extraintestinal manifestation
HR	Hazard ratio
IBD	Inflammatory bowel disease
OR	Odds ratio
PSC	Primary sclerosing cholangitis
SD	Standard deviation
SMD	Standardized mean difference
UC	Ulcerative colitis
