# Supplementary material for: Inflammatory Bowel Diseases Are Not Associated with an Increased Risk of Autoimmune Thyroiditis
Source: Med Sci (Basel). 2026 Jan 31;14(1):65. doi: 10.3390/medsci14010065 (PMC12921990; doi:10.3390/medsci14010065)
Supplement: Supplementary file 1 [file medsci-14-00065-s001.zip › medsci-4080929-supplementary.pdf]

Supplementary information

Table S1. Number of events (M. Hashimoto, Grave's disease) in individuals with and without IBD (Crohn's disease, Ulcerative colitis)

	IBD vs. no IBD		Crohn's disease vs. no IBD		Ulcerative colitis vs. no IBD	
Event / Cohorts	IBD	No IBD	Crohn's disease	No IBD	Ulcerative colitis	No IBD
M. Hashimoto						
Total	154	148	158	156	151	143
Age 18-34 years	140	205	116	198	164	212
Age 35-50 years	207	177	215	171	201	182
Age 51-64 years	148	136	149	147	146	128
Age 65+ years	102	67	138	81	82	59
Female	252	246	243	251	258	241
Male	64	59	70	58	60	60
Grave's disease						
Total	45	35	45	33	45	35
Age 18-34 years	28	37	23	35	33	40
Age 35-50 years	28	38	43	35	16	40
Age 51-64 years	54	33	44	35	61	32
Age 65+ years	80	29	88	27	75	31
Female	72	52	73	50	71	53
Male	21	19	18	17	24	20
